# A Weather-Based Prediction Model of Malaria Prevalence in Amenfi West District, Ghana

**DOI:** 10.1155/2017/7820454

**Published:** 2017-01-31

**Authors:** Esther Love Darkoh, John Aseidu Larbi, Eric Adjei Lawer

**Affiliations:** ^1^Department of Theoretical and Applied Biology, College of Science, Kwame Nkrumah University of Science and Technology, Kumasi, Ghana; ^2^Department of Biodiversity Conservation and Management, Faculty of Natural Resources and Environment, University for Development Studies, Nyankpala Campus, Tamale, Ghana

## Abstract

This study investigated the effects of climatic variables, particularly, rainfall and temperature, on malaria incidence using time series analysis. Our preliminary analysis revealed that malaria incidence in the study area decreased at about 0.35% annually. Also, the month of November recorded approximately 21% more malaria cases than the other months while September had a decreased effect of about 14%. The forecast model developed for this investigation indicated that mean minimum (*P* = 0.01928) and maximum (*P* = 0.00321) monthly temperatures lagged at three months were significant predictors of malaria incidence while rainfall was not. Diagnostic tests using Ljung-Box and ARCH-LM tests revealed that the model developed was adequate for forecasting. Forecast values for 2016 to 2020 generated by our model suggest a possible future decline in malaria incidence. This goes to suggest that intervention strategies put in place by some nongovernmental and governmental agencies to combat the disease are effective and thus should be encouraged and routinely monitored to yield more desirable outcomes.

## 1. Introduction

Malaria transmission predominantly occurs in tropical and subtropical areas where* Anopheles* mosquitoes can survive and multiply. Vectors of the disease are geographically specific. Currently, there are 104 malaria endemic countries and approximately half the world's population are at risk of infection [1]. Though malaria has been successfully eliminated in temperate regions of the world, the disease is on the rise in Africa [2]. In Ghana, malaria is a principal public health problem which plagues all segments of the society [3]. As a result, the government in line with the Millennium Development Goals implemented a national malaria control program (NMCP) to help reduce malaria morbidity and mortality. It is important to note that malaria vector control interventions using long lasting insecticide treated nets (LLINs) and indoor residual spraying (IRS) have been promoted in the country since 1998 and 2006, respectively. However, due to some challenges encountered with the previous intervention (i.e., 1998-LLIN), the country started a door-to-door distribution and hang up campaign in 2010 (i.e., 05/2010–10/2012) through the NMCP [4]. To maintain universal coverage, it again pioneered a mixed model distribution mechanism using antenatal care, schools, and work places among others in 2013.

Generally, environmental factors have contributed significantly to malaria prevalence and thereby affected its distribution, seasonality, and transmission intensity [5]. Although several other factors account for its occurrence and incidence, the disease seems to have a significant association with climatic variables [6]. Malaria has been identified as the most climate sensitive disease [7]; hence changes in temperature, rainfall, and humidity due to climatic change are expected to influence malaria prevalence directly (modifying the behavior and geographical distribution of malaria vectors as well as changing the length of the cycle of the parasite) and indirectly (changing ecological relationships important to the organisms).

Several studies have attempted to predict epidemics by use of climatic variables that are predictors of malaria transmission potential. In spite of this, little consensus has emerged about the relative importance and predictive value of different factors [8–10]. For instance, Kassa and Beyene [11] found an increase in malaria cases during heavy rainfall while other years with excessive rainfall accounted for reduced malaria incidences. In Ghana, malaria prevalence is affected by flooding and warmer temperatures which cause about seven months of intense malaria transmission beginning in April/May to September [12]. Mbogo et al. [13] also found variation in the relationship between mosquito population and rainfall in different districts of Kenya and attributed these changes to environmental heterogeneity. In addition, Thomson et al. [14] indicated that malaria occurrence was influenced by rainfall and temperature while Ndiaye et al. [15] found a very high correlation between malaria mortality and rainfall but no impact from temperature or humidity.

According to Christiansen-Jucht et al. [16], temperature and precipitation alter vector biting rate, duration of their gonotrophic cycles, fecundity, and development of immature mosquitoes and adults. It has been shown that the development of* Anopheles gambiae* is strongly impeded at low temperatures and as such its larvae stops development below 16°C and dies at a temperature below 14°C [17]. Thus, for adult mosquitoes, the rate at which they feed on human blood is determined by ambient temperature. Human blood fed on by mosquitoes every 4 days at 17°C consequently speeds up parasite sporogonic development leading to an increased transmission efficacy [16, 17]. Plasmodium species are also sensitive to temperature such that parasites-inhabiting female mosquito is reduced when external temperature is high (above 34°C). In spite of this, the effect of temperature is greatest on transmission at lower (i.e., 17–21°C) temperature [17].

Knowledge of the dynamic structure underlying data collected at regular time intervals (i.e., time series data) can be useful in generating forecasts through modeling. This can be achieved either by self-projecting or cause-effect modeling techniques [18]. Self-projecting approaches produce predictions using only time series data of the activity to be forecasted while the latter relies on relationships between the time series to be forecasted and one or more series that influence it. Several models for forecasting time series data have been developed (e.g., exponential, ARIMA, GARCH, and artificial intelligence) and applied to diverse fields of study including medicine over the past decades [19]. Available literature on malaria forecasting shows that quite a large number of studies rely on the cause-effect approach where covariates such as temperature, vegetation (NDVI), and/or rainfall among others are included in malaria models [20–24]. Nonetheless, forecasts generated using any of these stochastic models (i.e., time series analysis) can play significant roles in disease management or control strategies. Therefore, understanding the impact of climatic variables on malaria prevalence is important for effective policy and management decisions locally and globally. Hence, this study seeks to employ a time series analysis model to predict malaria incidence in the Amenfi West District of Ghana using weather (i.e., rainfall and temperature) as exogenous inputs.

## 2. Materials and Methods

### 2.1. Study Area

**Figure 1:**
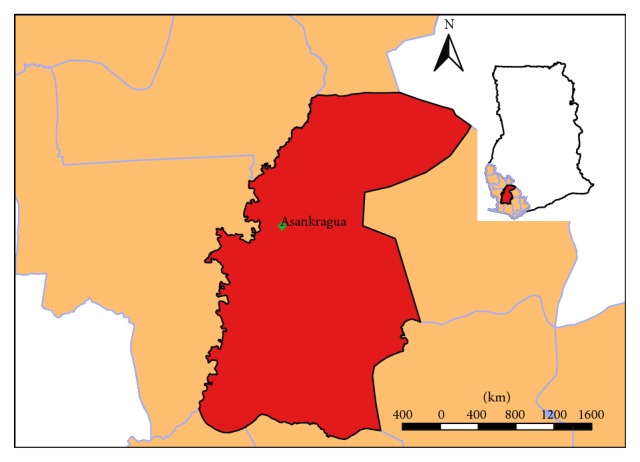
Map of Ghana showing study area in Amenfi West District.

The study was carried out at Asankrangwa Catholic Hospital in the Amenfi West District of the Western Region, Ghana (Figure 1). It is the largest health facility (district hospital) serving both as the first consultation point and as a referral centre for about 13 health care facilities in the district. This hospital was chosen since most of the 13 health care facilities are community clinics and lack laboratory services as well as past records of confirmed malaria cases from 2002 to 2015. The district's geographical coordinates are 5°48′18′′N and 2°26′25′′. It is characterized by a bimodal rainfall regime, with the major season occurring from March to July and the minor season from September to mid-December. A dry spell is experienced between late December and February [25].

### 2.2. Data Collection

Monthly data of confirmed malaria cases as well as rainfall and temperature from 2002 to 2015 were obtained from the district hospital and the meteorological office, respectively. Until 2010, detection of malaria parasites in the study area during the period under consideration was done microscopically on stained thick and thin blood smears. However, for the remainder of the period, detection of malaria in the health facility was done using both Rapid Diagnostic Tests (RDT) and microscopy.

### 2.3. Data Analysis

A preliminary analysis was first conducted on the dataset to describe and investigate the nature of the trend characterizing the number of malaria cases in the district. In order to determine the trend, linear, quadratic, log-linear, and log-quadratic regression models were fitted and compared. Afterwards, monthly changes in the number of malaria cases were estimated using the selected time trend and a set of seasonal dummy variables (i.e., seasonal categories based on months). The intercept was not included in the model to avoid dummy variable trap. To show differential effects in terms of percentage change, Halvorsen and Palmquist [26] method of interpreting differential coefficients in semilogarithmic equations was used (i.e., [*e*^*γ*^ − 1] × 100, where *γ* is the coefficient of the dummy variable). The subsections below provide information on statistical approaches used for subsequent analyses in this research.

#### 2.3.1. Time Series Modeling

In this study, an autoregressive integrated moving average (ARIMA) approach popularly referred to as Box-Jenkins methodology was employed for modeling malaria incidence. The model takes into account past values of the data, prediction errors, and a random term [27]. Specifically, an ARIMAX model which is an extension of ARIMA modeling was employed. This model uses exogenous inputs which consist of external variables (predictors) to help explain the behavior of a dependent variable. Thus, *Y*_*t*_ time series of malaria incidence can be explained by *X* predictor variables such as rainfall and/or temperature. Since malaria incidence is influenced by seasonality, monthly number of malaria cases will show periodic or cyclic patterns. Thus, an ARIMA model may be unable to capture such seasonal behavior. Hence, a seasonal ARIMA (SARIMA) model was proposed for use in this study. The general equation of the SARIMAX model denoted by ARIMA(*p*, *d*, *q*)(*sp*, *sd*, *sq*)*X* which is an extension of the nonseasonal ARIMA(*p*, *d*, *q*)*X* can be written as

(1) ∅pBΦspB∇d∇ssdYt−∑i=1rciXi−μ=θqBΘsqBAt,

where *Y*_*t*_ is the observation of a time series at time* t*;* A* is a time series of *n* white noise observations;* B* is the delay operator; that is, *BY*_*t*_ = *Y*_*t*−1_; ∇ = 1 − *B* is the differentiating operator; that is, ∇*Y*_*t*_ = (1 − *B*)*Y*_*t*_ = *Y*_*t*_ − *Y*_*t*−1_; *μ*, *∅*_*p*_, *θ*_*q*_, respectively, represent the mean and parameter vectors of the autoregressive and moving average terms; Φ_*sp*_(*B*) is the *sp* seasonal autoregressive polynomial; Θ_*sq*_(*B*) is the *sq* seasonal moving average polynomial; and *c*_*i*_ are the regressors' coefficients.

#### 2.3.2. Model Selection, Diagnostics, and Forecast Errors

Diagnostic tests on the developed models were done using Ljung-Box and ARCH-LM tests. The Ljung-Box test was performed to determine whether model residuals were random and independent over time (i.e., no serial correlation) while the ARCH-LM test was employed to check for homoscedasticity of model residuals (i.e., if variances are constant over time). In order to evaluate the performance of the final model, measures of forecast error such as Mean Percent Error (MPE), Mean Absolute Error (MAE), Root Mean Square Error (RMSE), Mean Absolute Percent Error (MAPE), and Mean Absolute Scaled Error (MASE) were computed. For each forecast error estimated lower values are preferred. Mathematical details of the aforementioned statistics and how they are computed are provided elsewhere [28–32]. All analyses were performed using Gretl and R statistical software, specifically “forecast” package. The choice of model (either ARIMAX or SARIMAX) was automatically estimated and selected using information criteria approaches (i.e., Bayesian information criterion—BIC).

## 3. Results

### 3.1. Preliminary Analysis

Descriptive statistics of malaria incidence/cases, rainfall, and temperature (minimum, maximum, and average) recorded for the study area are presented in Table 1. Minimum and maximum values of malaria cases observed during the period of investigation were 192.00 and 2112.00, respectively. Rainfall had minimum and maximum values of 0.00 mm and 1022.20 mm while that of average temperature was 23.50°C and 31.00°C, respectively.

**Figure 2:**
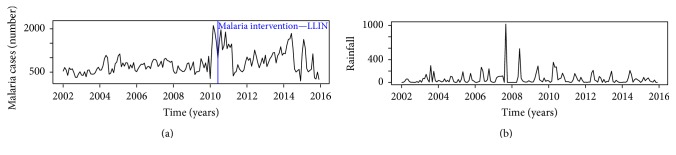
Time series plot—malaria cases showing the LLIN intervention in May 2010 (a) and rainfall data (b).

**Figure 3:**
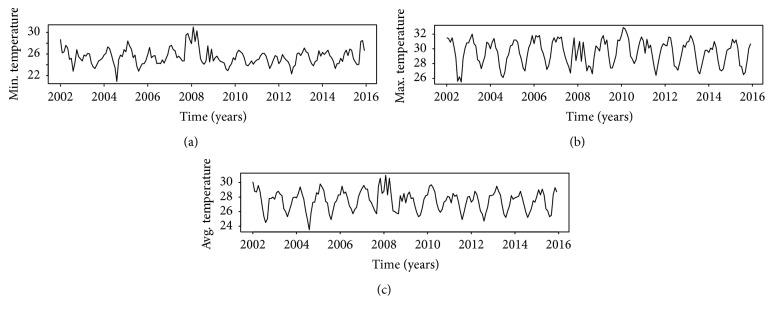
Time series plot of temperature ((a) min.; (b) max.; and (c) avg.).

Time series plots of data (malaria, rainfall, and temperature) gathered for the research are shown in Figures 2 and 3. The series plot of rainfall revealed that the amount of rains was constant over time with some heavy downpours observed in late 2007 and early 2008. Interestingly, a cursory examination of Figure 1 showed that high rainfall values within that period did not result in increased malaria incidence instantly. Both rainfall and temperature plots exhibited cyclic/periodic patterns which is an indication of seasonal dependency.

**Figure 4:**
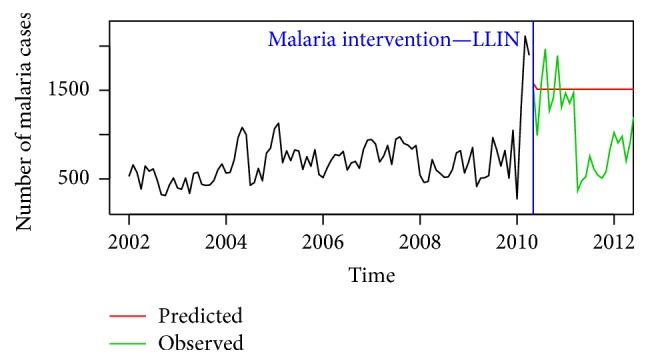
Plot of preintervention forecast compared to observed malaria cases.

Figure 4 shows forecasts generated using data before the 2010 LLIN intervention based on an ARIMA(0,1, 2) model. From the figure, the preintervention forecast indicates a high malaria incidence rate compared to the actual/observed values after the LLIN intervention in the study area.

An investigation of the nature of the trend characterizing malaria incidence was subsequently performed and the results are presented in Table 2. Based on the selection criteria, a log-linear trend model was suitable for malaria incidence, thus confirming the slightly increasing trend observed in the time series plot. In order to investigate the effect of seasonality, the logarithmic transformed malaria incidence was regressed on the linear trend and seasonal dummies. The results indicate that the model is significant at the 5% significance level (*F*-statistic = 3130, *P* value = 0.00; *R*-square = 99.62%; and adjusted *R*-square = 99.95%). To quantify the monthly changes, the transformed malaria incidence data was first differenced and again regressed on the trend and seasonal dummy variables (Table 3). From Table 3, it can be seen that malaria incidence at the study area decreased at about 0.35% per year. Also, the month of November has approximately 21% more malaria cases than the others suggesting that it is the month in which the highest number of malaria cases occurs. The least number of malaria cases occurs in the month of September with a decreased effect of about 14%.

### 3.2. Time Series Analysis of Malaria Incidence

**Figure 5:**
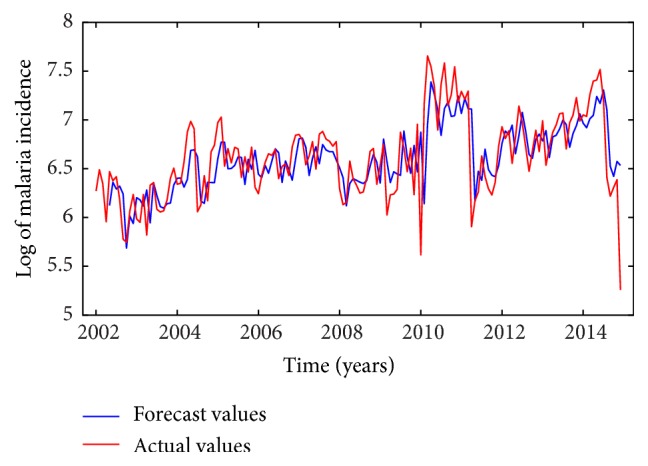
Plot of in-sample forecasts compared to training set data.

**Figure 6:**
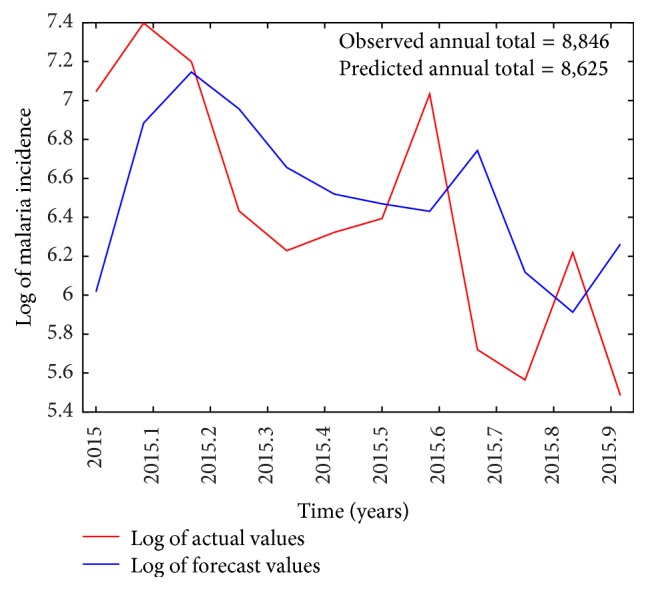
Plot of out-of-sample forecasts compared to the test set.

Following the time series analysis procedure outlined in the previous section, an ARIMA(1,1, 1) model was selected for modeling malaria incidence in the study area. The predictors included in the final model were minimum and maximum temperatures lagged at three months since the effect of rainfall (*P* = 0.7205) was not significant. Thus, the forecast model includes maximum and minimum temperature in the current month and the two months before that. Parameter estimates of the final model are provided in Table 4. From the table, a unit rise in maximum temperature in a given month will result in an associated increase of malaria cases three months later. On the other hand, a unit rise in minimum temperature will lead to a decline of malaria cases three months later. Figures 5 and 6 show plots of the original time series with the forecasted values based on the developed ARIMAX model. It can be observed that both the in-sample and the out-of-sample forecasts fitted the observed/actual values quite well. Forecast errors of the developed model based on the in-sample and out-of-sample forecasts are given in Table 5.

The results indicate that all forecast error values estimated were low and that the model was accurate since MAPE values for both training and test sets were less than 10%. Subsequently, the model was diagnosed using the statistics provided in Table 6. The Ljung-Box test yielded a *P* value of 0.7799 indicating that the model was free from serial correlation while the ARCH-LM (*P* = 0.07685) test revealed that model residuals were homoscedastic.

**Figure 7:**
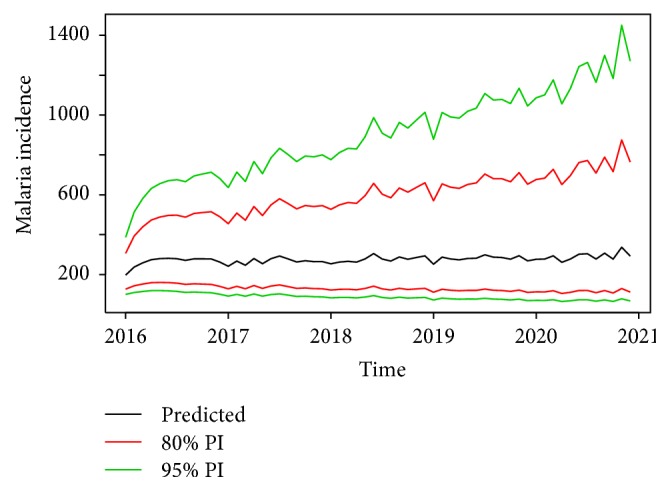
Forecast of malaria Incidence (i.e., based on back-transformed output) with 80% and 95% prediction intervals (PI).

A forecast plot generated by the model from 2016 to 2020 is shown in Figure 7. Point forecasts (predicted values) revealed that malaria incidence will fluctuate around 200 patients per month with an upper 95% prediction interval (PI) ranging from 400 to 1400 patients for the forecast period (85% PI: 306–875 patients). Thus, compared to past malaria incidence (2002–2015), both upper PIs (80% and 95%) show a nonincreasing trend in malaria incidence for the forecast period considered (i.e., observed/past min. and max. values were 192.0 and 2112.0 cases, resp.). However, minimum values (i.e., lower PIs) of malaria cases forecasted for the study area were 65 and 105 patients per month based on 95% and 80% PI, respectively.

## 4. Discussion

According to Jaffar et al. [33], peak in morbidity and mortality of malaria is generally obtained in the rainy season. This rise could be attributed to higher levels of parasitaemia in the rainy season than in the dry season as was reported by Greenwood and Pickering [34]. Similarly, our findings indicated that the highest monthly effect in terms of malaria incidence occurred in the rainy season (i.e., major season, May = ~9%; minor season, November = ~21%). Generally, onset of the two rainy seasons did not result in substantial growths in recorded malaria cases until after the second month specifically, in the third. This is corroborated by Alemu et al. [6] who asserted that climatic factors may not have instantaneous effects on malaria incidence rather they may have lagged effects. This may be because of delayed oviposition by the female mosquito due to heavy or erratic rainfall [35] common to the study area, as moderate rainfall and heat produce suitable habitat for oviposition [36, 37].

Furthermore, the findings of this study which are consistent with others (e.g., [38]) suggest that rainfall (even when lagged) did not significantly influence the predictive ability of the forecast model developed for the study area. Contrarily, other works have revealed a strong positive association between incidence of malaria and rainfall [6, 11, 15]. Unarguably, though rainfall creates breeding sites for mosquitoes [15], we believe that excessive amounts may destroy their breeding grounds, thereby causing reduced numbers of disease vectors as was reported elsewhere [39, 40]. Also, the general lack of consistency in weather variables especially rainfall in predicting malaria incidence could be attributed to complex interactions (i.e., between weather variables and disease transmission) that affect vector recruitment, abundance and survival, and parasite maturation [10]. These interactions as well as lagged effects could possibly explain the high incidence observed in the month of February (~12%) after the end of the minor rainy season in mid-December.

Some studies have reported the importance of temperature in malaria transmission or prediction models [41, 42]. Basically, research has shown that there is a nonlinear relationship between extrinsic incubation period (EIP) and temperature. EIP is very temperature sensitive in that small changes in the latter may result in significantly large effects on malaria transmission. Thus, as temperature increases, mosquitoes become infectious quickly due to a shortened EIP and vice versa [43–46]. Hence, the, respectively, negative and positive association of minimum and maximum temperatures with malaria incidence is somewhat suggestive of the nonlinear relationship between the two variables (i.e., temperature and malaria) as mentioned earlier. Teklehaimanot et al. [10] noted that, at low temperatures, complete development of mosquitoes at larval and pupal stages was delayed. In addition, Beck-Johnson et al. [47] revealed that recruitment dynamics (age structure) of mosquitoes were temperature dependent and thus exhibited a nonlinear behavior. Even though their study revealed that abundance was the largest across the 20–30°C temperature range, it mostly peaked around mid-20°C. Although the decline in abundance was not significantly large, they recorded lower numbers when temperatures were approaching 20°C and lower. Also, they reported a decrease in longevity of mosquitoes at temperatures above 32°C. Essentially, the significance of both minimum and maximum monthly temperatures in our predictive models could be attributed to the consistency of these values falling within the range suitable for a viable sexual cycle of the parasite in the mosquito. We can also safely infer that increased temperature at optimum make mosquitoes feed on blood meal at shorter intervals [46] possibly due to accelerated digestion [17] and biting rates [48], thus influencing malaria transmission.

A number of studies have documented some potential predictors of malaria such as population growth [49], economic hardship [2], and poor environmental sanitation and housing conditions [50]. For instance, high temperatures may cause people to sleep outside or inside their rooms without covering themselves, thereby increasing the risk of mosquito bite at night [39] because of problems attributed to finance or housing conditions. Moreover, due to variation in incubation period among* Plasmodium* species, it may be possible that species responded differently to the extent of temperature in the study area [51]. Hence, we do not neglect all these probable limitations and the impacts they could have had on our findings. Nonetheless, our forecast of malaria incidence till 2020 suggests a future decline in malaria cases compared to previously reported years. Also, the number of malaria cases following the 2010 intervention decreased compared to the preintervention forecast. Our findings further suggested that malaria incidence in the study area decreased annually at approximately 0.35% over the past years. This is undoubtedly due to the effective intervention strategies put in place by the country's NMCP to combat the disease over the years [52].

## 5. Conclusion

The findings of this study present a clear association of temperature and malaria incidence and how malaria cases are likely to reduce in the future based on our forecast model. We, however, cannot ignore the fact that other contributory factors may modulate malaria prevalence in the study district. Hence, it is recommended that future works on malaria incidence in the district should incorporate population size, intervention strategies, and vegetation (NDVI) among others to help improve its predictive accuracy. Also, various ongoing interventions such as sleeping in insecticide treated nets (i.e., LLINs), proper drainage systems, and sanitation practices should be continued/encouraged to help curb the disease.

## Figures and Tables

**Table 1:** Descriptive statistics of study variables.

Variable	Mean	Minimum	Maximum	CV (%)
Malaria incidence	809.800	192.000	2112.000	45.730
Rainfall	60.820	0.000	1022.200	181.640
Min. temperature	25.455	20.900	31.000	5.950
Max. temperature	29.549	25.500	32.900	5.660
Avg. temperature	27.507	23.500	31.000	5.090

Min. = minimum, max. = maximum, and avg. = average.

**Table 2:** Trend analysis of malaria incidence.

Model	AIC	AICC	BIC
Linear	1967.043	2445.953	2455.178
Quadratic	1981.416	2460.326	2469.551
Log-linear	−287.694^*∗*^	191.215^*∗*^	200.441^*∗*^
Log-quadratic	−286.082	192.827	202.053

*∗*: means best model based on selection criteria.

**Table 3:** Estimates of log first differenced series with monthly effects.

Variable	Coefficient	Standard error	Percent effect
January	0.04697	0.10828	4.80884
February	0.11713	0.10439	12.42660
March	−0.00834	0.10437	−0.83026
April	−0.13063	0.10435	−12.24537
May	0.08189	0.10435	8.53359
June	0.00370	0.10434	0.37096
July	−0.00751	0.10434	−0.74837
August	−0.08839	0.10434	−8.45940
September	−0.14806	0.10435	−13.76162
October	0.02261	0.10435	2.28687
November	0.18997	0.10437	20.92168
December	−0.13216	0.10439	−12.37984
Trend	−0.00351	0.00754	−0.35082

*Note*. Effect of January = (*e*^0.04697^ − 1) × 100%.

**Table 4:** Parameter estimates of the developed model.

Parameter	Estimate	Std. error	*Z*-statistic	*P* value
ar1 (*ϕ*)	0.41283	0.15768	2.61820	0.00884
ma1 (*θ*)	−0.85977	0.11499	−7.47670	0.00000
Max. temperature	0.07599	0.02579	2.94650	0.00321
Min. temperature	−0.06653	0.02843	−2.34010	0.01928

**Table 5:** Estimated forecast errors for the developed model.

Variable	ME	MSE	MAPE	RMSE	MAE	Theil's *U*
Training set	0.00966	0.09953	3.48070	0.31548	0.22677	0.90767
Test set	−0.08955	0.35334	8.09080	0.59442	0.50688	0.91227

**Table 6:** Diagnostic tests on model residuals.

Method	df	Chi-square	*P* value
Ljung-Box test	24	18.46500	0.77990
ARCH-LM test	24	34.45800	0.07685

## References

[1] Kiszewski A., Mellinger A., Spielman A., Malaney P., Sachs S. E., Sachs J. (2004). A global index representing the stability of malaria transmission. *The American Journal of Tropical Medicine and Hygiene*.

[2] Sachs J., Malaney P. (2002). The economic and social burden of malaria. *Nature*.

[3] World Health Organization (2009). *Malaria Report in Ghana*.

[4] Gakpey K., Baffoe-Wilmot A., Malm K., Dadzie S., Bart-Plange C. (2016). Strategies towards attainment of universal coverage of long lasting insecticide treated nets (LLINs) distribution: Experiences and lessons from Ghana. *Parasites and Vectors*.

[5] Snow R. W., Craig M., Deichmann U., Marsh K. (1999). Estimating mortality, morbidity and disability due to malaria among Africa's non-pregnant population. *Bulletin of the World Health Organization*.

[6] Alemu A., Abebe G., Tsegaye W., Golassa L. (2011). Climatic variables and malaria transmission dynamics in Jimma town, South West Ethiopia. *Parasites and Vectors*.

[7] Roll Back Malaria Climate change and Malaria. http://www.google.com.gh/search?q=climate+change+and+malaria+2015.pdf&client=ms-opera-mini-android&channel=new&gws_rd=cr&ei=tUzUVrytJ8uIaMHmiMAF.

[8] Woube M. (1997). Geographical distribution and dramatic increases in incidences of malaria: consequences of the resettlement scheme in Gambela, SW Ethiopia. *Indian Journal of Malariology*.

[9] Abeku T. A., van Oortmarssen G. J., Borsboom G., de Vlas S. J., Habbema J. D. F. (2003). Spatial and temporal variations of malaria epidemic risk in Ethiopia: factors involved and implications. *Acta Tropica*.

[10] Teklehaimanot H. D., Lipsitch M., Teklehaimanot A., Schwartz J. (2004). Weather-based prediction of *Plasmodium falciparum* malaria in epidemic-prone regions of Ethiopia I. Patterns of lagged weather effects reflect biological mechanisms. *Malaria Journal*.

[11] Kassa A. D., Beyene B. B. (2014). Climate variability and malaria transmission—fogera district, Ethiopia, 2003–2011. *Science Journal of Public Health*.

[12] Akpalu W., Codjoe S. N. A. (2013). Economic analysis of climate variability impact on malaria prevalence: the case of Ghana. *Sustainability (Switzerland)*.

[13] Mbogo C. M., Mwangangi J. M., Nzovu J. G. (2003). Spatial and temporal heterogeneity of *Anopheles mosquitoes* and *Plasmodium falciparum* transmission along the Kenyan coast. *The American Journal of Tropical Medicine and Hygiene*.

[14] Thomson M. C., Mason S. J., Phindela T., Connor S. J. (2005). Use of rainfall and sea surface temperature monitoring for malaria early warning in Botswana. *The American Journal of Tropical Medicine and Hygiene*.

[15] Ndiaye O., le Hesran J.-Y., Etard J.-F. (2001). Variations climatiques et mortalité attribuée au paludisme dans la zone de niakhar, sénégal de 1984 À 1996. *Santé*.

[16] Christiansen-Jucht C., Parham P. E., Saddler A., Koella J. C., Basáñez M.-G. (2014). Temperature during larval development and adult maintenance influences the survival of Anopheles gambiae s.s. *Parasites & vectors*.

[17] Afrane Y. A., Githeko A. K., Yan G. (2012). The ecology of Anopheles mosquitoes under climate change: case studies from the effects of deforestation in East African highlands. *Annals of the New York Academy of Sciences*.

[18] Lohani A. K., Kumar R., Singh R. D. (2012). Hydrological time series modeling: a comparison between adaptive neuro-fuzzy, neural network and autoregressive techniques. *Journal of Hydrology*.

[19] Lawer E. A. (2016). Empirical modeling of annual fishery landings. *Natural Resources*.

[20] Abeku T. A., De Vlas S. J., Borsboom G. (2002). Forecasting malaria incidence from historical morbidity patterns in epidemic-prone areas of Ethiopia: a simple seasonal adjustment method performs best. *Tropical Medicine and International Health*.

[21] Gomez-Elipe A., Otero A., van Herp M., Aguirre-Jaime A. (2007). Forecasting malaria incidence based on monthly case reports and environmental factors in Karuzi, Burundi, 1997–2003. *Malaria Journal*.

[22] Wangdi K., Singhasivanon P., Silawan T., Lawpoolsri S., White N. J., Kaewkungwal J. (2010). Development of temporal modelling for forecasting and prediction of malaria infections using time-series and ARIMAX analyses: a case study in endemic districts of Bhutan. *Malaria Journal*.

[23] Krefis A. C., Schwarz N. G., Krüger A. (2011). Modeling the relationship between precipitation and malaria incidence in children from a holoendemic area in Ghana. *American Journal of Tropical Medicine and Hygiene*.

[24] Zinszer K., Kigozi R., Charland K. (2015). Forecasting malaria in a highly endemic country using environmental and clinical predictors. *Malaria Journal*.

[25] Ministry of Local Government and Rural Development Amenfi West. http://www.ghanadistricts.com/About-District-Details.aspx?distID=198&distName=Amenfi%20West.

[26] Halvorsen R., Palmquist R. (1980). The interpretation of dummy variables in semilogarithmic equations. *The American Economic Review*.

[27] Box G. E., Jenkins G. M. (1970). *Time Series Analysis, Forecasting and Control*.

[28] Ljung G. M., Box G. E. P. (1978). On a measure of lack of fit in time series models. *Biometrika*.

[29] Engle R. F. (1982). Autoregressive conditional heteroscedasticity with estimates of the variance of United Kingdom inflation. *Econometrica. Journal of the Econometric Society*.

[30] Kwiatkowski D., Phillips P. C. B., Schmidt P., Shin Y. (1992). Testing the null hypothesis of stationarity against the alternative of a unit root: how sure are we that economic time series have a unit root?. *Journal of Econometrics*.

[31] Hyndman R. J., Koehler A. B. (2006). Another look at measures of forecast accuracy. *International Journal of Forecasting*.

[32] Shumway R. H., Stoffer D. S. (2011). *Time series analysis and its applications*.

[33] Jaffar S., Leach A., Greenwood A. M. (1997). Changes in the pattern of infant and childhood mortality in Upper River Division, The Gambia, from 1989 to 1993. *Tropical Medicine and International Health*.

[34] Greenwood B. M., Pickering H. (1993). A malaria control trial using insecticide-treated bed nets and targeted chemoprophylaxis in a rural area of The Gambia, west Africa. 1. A review of the epidemiology and control of malaria in The Gambia, west Africa. *Transactions of the Royal Society of Tropical Medicine and Hygiene*.

[35] Strickman D. (1983). Preliminary report of seasonal oviposition by Culex quinquefasciatus in San Antonio, Texas. *Mosquito News*.

[36] Reisen W. K., Hardy J. L., Presser S. B. (1992). Mosquito and arbovirus ecology in southeastern California, 1986–1990. *Journal of Medical Entomology*.

[37] Doggett S. L., Russell R. C., Clancy J., Haniotis J., Cloonan M. J. (1999). Barmah Forest virus epidemic on the south coast of New South Wales, Australia, 1994-1995: viruses, vectors, human cases, and environmental factors. *Journal of Medical Entomology*.

[38] Hamel M. J., Adazu K., Obor D. (2011). A reversal in reductions of child mortality in Western Kenya, 2003–2009. *American Journal of Tropical Medicine and Hygiene*.

[39] Nkurunziza H., Gebhardt A., Pilz J. (2010). Bayesian modelling of the effect of climate on malaria in Burundi. *Malaria Journal*.

[40] Huang T. F., Zhou S., Zhang S., Wang H., Tang L. (2011). Temporal correlation analysis between malaria and meteorological factors in Motuo County, Tibet. *Malaria Journal*.

[41] Loevinsohn M. E. (1994). Climatic warming and increased malaria incidence in Rwanda. *The Lancet*.

[42] Hoshen M. B., Morse A. P. (2004). A weather-driven model of malaria transmission. *Malaria Journal*.

[43] Ruiz D., Poveda G., Vélez I. D. (2006). Modelling entomological-climatic interactions of *Plasmodium falciparum* malaria transmission in two Colombian endemic-regions: contributions to a National Malaria Early Warning System. *Malaria Journal*.

[44] Paaijmans K. P., Read A. F., Thomas M. B. (2009). Understanding the link between malaria risk and climate. *Proceedings of the National Academy of Sciences of the United States of America*.

[45] Kim Y., Schneider K. A. (2013). Evolution of drug resistance in Malaria parasite populations. *Nature Education Knowledge*.

[46] Apinjoh T. O., Anchang-Kimbi J. K., Mugri R. N. (2015). The effect of Insecticide Treated Nets (ITNs) on *Plasmodium falciparum* infection in rural and semi-urban communities in the South West Region of Cameroon. *PLoS ONE*.

[47] Beck-Johnson L. M., Nelson W. A., Paaijmans K. P., Read A. F., Thomas M. B., Bjørnstad O. N. (2013). The effect of temperature on *Anopheles* mosquito population dynamics and the potential for malaria transmission. *PLoS ONE*.

[48] Lindblade K. A., Walker E. D., Onapa A. W., Katungu J., Wilson M. L. (2000). Land use change alters malaria transmission parameters by modifying temperature in a highland area of Uganda. *Tropical Medicine and International Health*.

[49] Van Lieshout M., Kovats R. S., Livermore M. T. J., Martens P. (2004). Climate change and malaria: analysis of the SRES climate and socio-economic scenarios. *Global Environmental Change*.

[50] Nkuo-Akenji T., Ntonifor N. N., Ndukum M. B. (2006). Environmental factors affecting malaria parasite prevalence in rural Bolifamba, South West Cameroon. *African journal of health sciences*.

[51] Blanford J. I., Blanford S., Crane R. G. (2013). Implications of temperature variation for malaria parasite development across Africa. *Scientific Reports*.

[52] United Nations Children's Fund UNICEF Ghana Fact Sheet Malaria; UNICEF: Accra, Ghana. http://www.unicef.org/wcaro/WCARO_Ghana_Factsheet_malaria.pdf.

